# Multifunctional irrigation mitigates heat stress, delays sugar accumulation and preserves berry composition in cv. Sangiovese (*Vitis vinifera* L.)

**DOI:** 10.3389/fpls.2026.1911234

**Published:** 2026-08-05

**Authors:** Gabriele Valentini, Alberto Zanini, Gianluca Allegro, Eleonora Cataldo, Alice Moffa, Filippo Casini, Ilaria Filippetti

**Affiliations:** 1 Department of Agricultural and Food Sciences, Alma Mater - University of Bologna, Bologna, Italy; 2 Department of Biotechnology - University of Verona, Verona, Italy; 3 Department of Agriculture, Food, Environment and Forestry (DAGRI), University of Florence, Florence, Italy

**Keywords:** climate change, drip irrigation, evaporative cooling, heat stress, wireless sensor network

## Abstract

**Introduction:**

Climate change increasingly threatens Mediterranean viticulture, as rising temperatures, increased radiation and reduced precipitation impose multiple stresses on grapevines, negatively affecting yield and grape composition. In red cultivars, these conditions often lead to a decoupling between technological and phenolic maturity, resulting in wines with higher alcohol content, reduced acidity and lower color intensity. In addition, sunburn damage further compromises both yield and berry quality. To address these challenges, a multifunctional irrigation system combining post-veraison drip irrigation with evaporative cooling of the fruit zone was developed.

**Methods:**

The system was tested during the 2024 and 2025 growing seasons in a flatland vineyard of Sangiovese (*Vitis vinifera* L.) in Bologna (Italy), by comparing vines subjected to multifunctional irrigation (MI) with rainfed controls (RC). Vine physiological responses, yield components, sunburn incidence and berry composition were monitored during ripening.

**Results and Discussion:**

The MI treatment resulted in consistent reductions in both air and cluster temperature, complete prevention of sunburn damage, and improved vine physiological status. In particular, across both seasons, MI vines exhibited improved leaf physiological performance, as reflected by increased stomatal conductance and net assimilation rates, with differences becoming more evident under moderate to severe water stress conditions. A significant Year x Irrigation (Y x I) interaction was observed for total soluble solids (TSS). In the drier and warmer 2025 season, MI vines showed lower TSS and higher titratable acidity at harvest compared to RC, indicating a significant but slight delay in technological maturity. Although anthocyanin concentration did not differ significantly between treatments at harvest, MI vines exhibited higher anthocyanin concentrations during ripening while the multifunctional irrigation system was active. These findings suggest that the combined irrigation–cooling strategy may allow harvest at lower sugar concentrations while preserving anthocyanin concentration, potentially contributing to a better balance between alcohol content and color intensity. In conclusion, the combined irrigation-cooling strategy can mitigate heat stress, delay sugar accumulation, and preserve both phenolic composition and acidity. However, its effectiveness depends on seasonal conditions and water input, and further studies are needed to evaluate its performance under more severe stress conditions, such as hillside vineyards, where water and heat stress are typically more pronounced.

## 1 Introduction

As temperatures continue to rise in the Mediterranean region as a consequence of climate change (IPCC, 2023; Ghaderpour et al., 2024; Baldan et al., 2025), the sustainability of traditional viticultural areas is increasingly threatened (Droulia and Charalampopoulos, 2021; Sodini et al., 2023). Higher temperatures advance grapevine (*Vitis vinifera* L.) phenology, including the onset of ripening, thereby forcing growers to harvest under hotter conditions (Faralli et al., 2024). Specifically, elevated temperatures during ripening induce a decoupling between technological and phenolic maturity in red grape cultivars, accelerating sugar accumulation while delaying or impairing phenolic development (Valentini et al., 2019; Vercesi et al., 2024). As a consequence, harvest dates are often advanced to a suboptimal level of phenolic maturity (Di Carlo et al., 2019; Van Leeuwen et al., 2022), resulting in wines with reduced color intensity, lower perceived quality (Parpinello et al., 2009; Sáenz-Navajas et al., 2011) and increased alcohol content (Van Leeuwen et al., 2024).

In parallel, climate change scenarios predict an increase in solar radiation intensity coupled with a decrease in precipitation frequency, leading to more frequent and severe drought events (IPCC, 2023; Alba et al., 2024). These concurrent stressors impair grapevine leaf physiological activity by reducing photosynthetic efficiency, stomatal conductance and carbon assimilation capacity (Flexas et al., 2014; Dinis et al., 2018; Valentini et al., 2024). Moreover, the interaction between high temperature and intense solar radiation enhances the incidence of berry sunburn, withering and tissue necrosis (Hulands et al., 2014), causing chemical alterations in grape berries (Allegro et al., 2024; Sangiorgio et al., 2025) that ultimately translate into modified wine composition (Šuklje et al., 2016; Allegro et al., 2019). Altogether, these factors contribute to significant yield losses in grapevine (Allegro et al., 2023). To mitigate these adverse effects, a range of agronomic strategies has been developed (Palliotti et al., 2014; Naulleau et al., 2021).

Row orientation and canopy management play a key role in limiting excessive grape exposure to solar radiation during the hottest hours of the day (Strack and Stoll, 2021). Shading the fruit zone using nets can effectively reduce sunburn damage, while whole canopy shading may delay ripening due to the reduced leaf photosynthetic performance (Oliveira et al., 2014; Palliotti et al., 2014). More recently, agrivoltaic systems have been proposed as an alternative approach to moderate radiation and temperature (Bonini et al., 2025b). In addition, vine vigor itself influences cluster microclimate and light exposure within the canopy, with consequences for grape composition and flavonoid accumulation (Romboli et al., 2017).

Foliar application of mineral-based products has also been proposed as an effective strategy to mitigate heat and radiation stress by increasing light reflectance and reducing fruit surface temperature (Teker, 2023; Allegro et al., 2024). In this context, kaolin and zeolite applications have been shown to partially alleviate the decoupling between technological and phenolic ripening (Valentini et al., 2021). Other approaches include the use of yeast-derived elicitors to stimulate anthocyanin biosynthesis in red grape cultivars (Villegas et al., 2016; Pastore et al., 2020).

A more effective long-term strategy to reduce vine thermal exposure is to shift ripening towards cooler periods of the season. Among the proposed approaches, minimal or semi-minimal pruning can modify vine balance and crop load, often increasing yield and, in some cases, delaying sugar accumulation or improving phenolic composition, depending on cultivar and growing conditions (Intrieri et al., 2011; Schäfer et al., 2021; Molitor et al., 2019). Other approaches aimed at delaying ripening are delayed winter pruning, late shoot trimming and late defoliation, although these techniques may involve trade-offs, particularly in terms of yield reduction or variable effects depending on genotype and timing (Martinez et al., 2019; Filippetti et al., 2011; Palliotti et al., 2014).

Irrigation can delay ripening and increase yield, although its effects on berry composition are highly variable and depend on timing, water amount and cultivar. Full-season irrigation generally delays total soluble solids (TSS) and anthocyanin accumulation while increasing yield (Theocharis et al., 2021). By contrast, irrigation applied from fruit set or at veraison shows more variable responses, depending on water supply (Caruso et al., 2023). In particular, post-veraison irrigation has been associated with contrasting effects on berry composition: in some cases, it delays anthocyanin accumulation and exacerbates the decoupling between technological and phenolic maturity (Theocharis et al., 2021), while in others it reduces both sugar and anthocyanin concentration while increasing yield (Allegro et al., 2023 and Allegro et al., 2024; Intrigliolo et al., 2016). Conversely, different responses have also been reported, including unchanged TSS and increased anthocyanin concentration under higher irrigation levels (Junquera et al., 2012). Despite the effectiveness of the above-mentioned strategies in mitigating heat and radiation stress or delaying ripening, none of them directly reduce the temperature experienced by clusters during ripening. Therefore, ultra-fine misting systems have been developed to evaporatively cool the canopy microclimate, reducing air temperature around clusters while limiting water consumption and leaf wetting (Matthias and Coates, 1986). Recently, Valentini et al. (2024) demonstrated that a fruit-zone cooling system capable of consistently lowering cluster temperature during ripening increased anthocyanin accumulation and yield in potted Sangiovese vines subjected to water restriction, while protecting berries from sunburn damage. This yield-protective effect was subsequently confirmed under field conditions in the non-irrigated white cultivar Pignoletto (Valentini et al., 2025).

Although post-veraison irrigation can increase berry size and induce dilution of berry constituents, particularly anthocyanins (Ojeda et al., 2002; Castellarin et al., 2007), its combination with fruit-zone cooling may represent a complementary strategy to delay sugar accumulation while mitigating heat stress. However, the interaction between water supply and berry composition remains complex and may lead to contrasting responses depending on water input and vine water status.

Based on these considerations, a multifunctional system combining post-veraison irrigation and fruit-zone cooling was designed and implemented in experimental vineyards at the University of Bologna. The aim of this study was to evaluate how a combined irrigation-cooling strategy affects vine water status, berry ripening dynamics and phenolic composition, in comparison with rainfed conditions, with particular emphasis on its potential to mitigate heat stress and limit the decoupling between technological and phenolic maturity.

## 2 Materials and methods

### 2.1 Plant materials and experimental design

The experiment took place in the 2024 and 2025 seasons in a vineyard planted in 2019 at the experimental station of the University of Bologna (Bologna, 44°32′ N, 11°22′ E). Plant material consisted of *Vitis vinifera* L. Sangiovese vines, clone TEA 10D, grafted onto 110 Richter rootstock, spaced at 1.0 m within a single row and 2.7 m between rows (oriented northeast to southwest), and trained to monolateral Guyot VSP system. Winter pruning left 12 buds per vine. In 2024, before veraison vines were uniformed via cluster thinning to a number of 16 bunches per vine. In 2025 the number of inflorescences was counted at BBCH53 and, seen as plants had uniform number of clusters, no thinning was performed. A randomized block design (RBD) was applied, consisting of 36 tagged vines divided into two treatments (18 vines per treatment). Six blocks of 3 vines each were considered per treatment (2 theses x 6 blocks x 3 plants = 36 total plants). The two treatments consisted in i) control vines that were rainfed (RC) and ii) vines subjected to multifunctional irrigation (MI), combining post-veraison drip irrigation and automated fruit-zone misting. Inter-rows were covered by ILMIX (S.I.S., San Lazzaro, BO, Italy) and mowed during the season to avoid excessive evapotranspiration. The under-row was managed mechanically, using an offset-type cultivator, tilling the soil periodically. Viticultural practices typical of the Emilia Romagna region were applied.

### 2.2 Characteristics of the multifunctional irrigation system

An automated multifunctional irrigation system was implemented on open field conditions, as an evolution of the system employed by Valentini et al. (2025). This system was composed of two distinct elements. The first component was an auto-compensating drip irrigation pipeline (drippers spaced 0.6 meter, delivering 3.8 L h^-^¹) installed 10 cm under the cordon, which would distribute water on a weekly basis so that 100% of the evapotranspirated water (ETc) would be given back to the plants. ETc was estimated using Manna software (Manna Irrigation, Gvat, Israel) according to Beeri et al. (2020). The second component of the system consisted of a misting line parallel to the drip irrigation, on which Super fogger green model emitters (Naandanjain Italy, Pomezia, Italy) were installed. The Super foggers (nominal discharge 11.2 L h^-^¹) were equipped with an anti-drip mechanism and were activated when temperature around canopy exceeded 35 °C (Valentini et al., 2025). Vines were spaced at 1.0 m x 2.7 m, and one Super fogger was installed per vine, resulting in approximately 3,700 emitters per hectare. The misting system operated in cyclic mode, with 5 min of activation followed by 15 min of inactivity, corresponding to an effective operating time of 15 min per hour. Under these conditions, each emitter delivered approximately 1.04 mm h^-^¹. Given the fine droplet size, prevailing evaporative conditions, and the location of emitters under the cordon, the nebulized water was assumed to evaporate before reaching the soil and to contribute mainly to evaporative cooling of the canopy (Matthias and Coates, 1986).

To control the system, two different electrovalves were managed by a control unit (iFarming S.r.l., Ravenna, RA, Italy). One electrovalve would control drip irrigation and the other the misting system. The control unit was wirelessly connected to temperature and humidity sensors inside the canopy, at fruit zone height. The system was activated on day of year (DOY) 206 (July 24^th^) during the 2024 season and on DOY 202 (July 21) during the 2025 season, corresponding approximatively to the veraison stage, and kept active until harvest.

### 2.3 Climatic data acquisition

Climatic data were recorded throughout the growing season using a weather station installed close to the experimental field (iFarming S.r.l., Ravenna, RA, Italy). To monitor the fruit-zone microclimatic conditions, each treatment was equipped with an independent monitoring unit consisting of a control unit, one temperature-relative humidity sensor positioned at fruit-zone height (approximately 30 cm above the main wire), and six thermocouples. The thermocouples were distributed among different vines within each treatment and inserted beneath the berry skin located on the east-, west-, and center-facing portions of the canopy to account for within-canopy variability.

An additional temperature and relative humidity sensor were installed in each treatment to continuously monitor air temperature, relative humidity and vapor pressure deficit (VPD) at fruit-zone height. In the MI treatment, the same sensor also provided the input signal for automatic activation of the misting system according to the predefined thresholds. All sensors recorded data at 10-minute intervals, and the measurements were automatically stored in a cloud-based system.

### 2.4 Physiological data

Physiological measurements were carried out after veraison on DOY 212, 220 and 248 in 2024, and DOY 202, 216 and 226 in 2025. Leaf net photosynthesis (A), leaf stomatal conductance (gs), and midday stem water potential (Ψ) were measured on each vine. Net photosynthesis and stomatal conductance were assessed on fully expanded leaves of the main shoot, between the 6^th^ and 10^th^ node, using a LI-6800 portable photosynthesis system (LI-COR, Lincoln, NE, USA) equipped with a broadleaf chamber under saturating light conditions (1500 µmol m^-^² s^-^¹). In both seasons, the same leaves used for gas exchange measurements were subsequently used to determine midday stem water potential (Ψ) using a model 1505D pressure chamber (PMS Instrument Company, Albany, OR, USA), following the method described by Turner and Long (1980).

### 2.5 Yield parameters and berry composition

On a weekly basis since veraison, samples of 30 randomly picked berries were taken from each vine of both treatments for measurement of quality parameters. Samples were weighted using a Mark electronic scale (Bel Engineering, Monza, Italy). Total soluble solid concentration (TSS) was measured from the juice extracted from the berries using a self-compensating Maselli R50 refractometer (Misure Maselli, Parma, Italy), while must pH and titratable acidity (TA) were determined using a Crison titrator (Crison Instruments, Barcelona, Spain). Once RC vines reached technological maturity, vines of both treatments were individually harvested and yield per vine was recorded. In detail, the number of harvested clusters per vine was recorded. Clusters completely desiccated by sunburn and unsuitable for yield determination were excluded from the count. Sunburn incidence on grape clusters was visually assessed following standard vineyard rating scales commonly used for sunburn and disease evaluation. For each vine, the percentage of cluster showing visible sunburn symptoms was calculated according to Equation 1.

(1) Sunburn Incidence (%)=(Number of sunburn affected clusters/Total number of assessed clusters)×100

For affected clusters, sunburn severity was visually estimated as the percentage of the exposed cluster area affected by necrotic or dehydrated berries, according to the method reported by Valentini et al. (2025). A hailstorm that affected the vineyard on August 27th, 2024 (DOY 240) prevented the assessment of sunburn damage. Therefore, sunburn incidence was not evaluated in 2024, and only the values recorded for the 2025 growing season are reported.

At harvest, on September 16^th^ 2024 (DOY 260) and September 3^rd^ 2025 (DOY 246), 30 berries per vine were harvested for analysis of technological parameters as previously reported. Both treatments were harvested on the same date in each season, reflecting standard commercial practice. Pruning weight per vine was measured during winter, at the end of each growing season, using a dynamometer.

### 2.6 Total anthocyanins assessment

Starting from veraison and at two-week intervals thereafter, 20 berries per vine were randomly collected. Total anthocyanins were extracted from the berry skins by soaking the peeled skins in 100 mL of methanol for 24 h. The extracts were then stored at −20 °C. The anthocyanins were quantified using high-performance liquid chromatography (HPLC) with a Waters 1525 system equipped with a diode array detector (DAD) and a reversed-phase RP18 column (250 × 4.6 mm, 5 µm) coupled with a pre-column (Phenomenex, Castel Maggiore, BO, Italy). Detection was performed at 520 nm, and quantification was carried out using an external calibration curve prepared with malvidin-3-glucoside chloride (Sigma-Aldrich, St. Louis, MO, USA), following the method described by Mattivi et al. (2006).

### 2.7 Statistical analysis

Data were analyzed using a three-way analysis of variance (ANOVA), with Year (Y) and Irrigation (I) as fixed factors. Sunburn incidence, expressed as percentage data, was analyzed separately using a generalized linear mixed model (GLMM) implemented in the lme4 package in R (R Foundation for Statistical Computing, Vienna, Austria). Sunburn severity was analyzed after Bliss angular transformation by one-way ANOVA considering only vines exhibiting sunburn symptoms. Differences between treatments were considered significant at P ≤ 0.05.

## 3 Results

### 3.1 Seasonal weather conditions

The two growing seasons under study (2024 and 2025) were characterized by markedly different climatic conditions. The 2024 season was both hot and exceptionally wet, whereas 2025 was generally hot, with particularly high temperatures in the early season followed by milder conditions during the later stages. In detail, the 2024 growing season (**Table 1**) exhibited an unusual weather pattern for the study area, mainly due to the distribution of rainfall. Contrary to typical conditions, where summer months are relatively dry, July, August and September were the wettest months of the year, with precipitation exceeding 190 mm in August and 180 mm in September, including approximately 70 mm before harvest. These months are critical for grapevine development, particularly for cv. Sangiovese, as they encompass the ripening period. At the same time, July and August were characterized by intense heatwaves, with mean maximum temperatures exceeding 33 °C and mean minimum temperatures reaching or exceeding 20 °C in August. Additionally, a severe hailstorm on August 27^th^ (DOY 240) caused damage to both canopy and fruit.

**Table 1:** Meteorological data recorded during the 2024 and 2025 growing seasons in Cadriano (BO).

Year	2024				2025			
	Tavg (°C)	Tmin (°C)	Tmax (°C)	Rainfall (mm)	Tavg (°C)	Tmin (°C)	Tmax (°C)	Rainfall (mm)
April	14.00	8.16	20.35	28.49	14.8	8.66	20.83	54.60
May	17.63	11.61	23.58	55.48	18.64	11.62	24.95	32.80
June	23.16	16.55	29.63	42.60	26.52	18.5	33.32	19.20
July	27.09	19.44	33.87	79.40	25.51	18.5	32.1	61.80
August	26.51	20.01	33.86	192.40	24.99	18.53	31.79	33.20
September	20.43	15.46	25.94	183.00	21.08	14.93	27.55	64.2

Monthly mean air temperature (Tavg), mean minimum (Tmin) and maximum (Tmax) air temperatures, and cumulative rainfall are reported.

The 2025 growing season (**Table 1**) was also warm but showed a different thermal pattern. Temperatures were particularly high from April to June, with mean values 1.73 °C higher than in the same period of 2024, while the second half of the season (July-September) was relatively cooler, although still affected by occasional heatwaves. Mean minimum temperatures rarely exceeded 18.5 °C, in contrast to 2024, when they reached up to 20 °C during peak heat periods. Rainfall in 2025 was generally in line with typical conditions for the area, except for July (61.8 mm), while most September precipitations occurred after harvest. In comparison, 2024 was substantially wetter, with more than double the total rainfall recorded in 2025.

### 3.2 Water supply from drip irrigation and misting system

The water balance between veraison and harvest differed markedly between the two seasons. In 2024, rainfall was the dominant water input, with 236.8 mm recorded during the ripening period. As a consequence, rainfed vines (RC) accumulated only a limited water deficit (34.5 mm relative to seasonal crop evapotranspiration, ETc). To compensate for this deficit, the multifunctional system supplied 35 mm of water through the drip irrigation line in the MI treatment. In contrast, the 2025 season was considerably drier, with only 87.8 mm of rainfall during the same period. Seasonal evapotranspiration was also lower (150 mm in 2025 compared to 271 mm in 2024), but this was insufficient to compensate for the reduced precipitation. Consequently, drip irrigation played a more important role in vine water supply. A total of 67 mm of water was supplied through the drip irrigation system in MI vines, resulting in a near-balanced water budget (+4 mm), whereas RC vines experienced a substantial water deficit (-63 mm).

The misting system, combined with the drip irrigation line, was active from veraison to harvest, with different frequencies in the 2024 and 2025 seasons. In 2024, activations were concentrated around the onset of veraison and reached a maximum of 111 events, corresponding to 38.3 mm of water applied to the fruit zone. In 2025, the number of activations was lower (69 events), resulting in 23.84 mm of water distributed. Overall, the multifunctional system supplied approximately 73 mm of water in 2024 (35 mm by drip irrigation and 38.3 mm by misting) and 91 mm in 2025 (67 mm by drip irrigation and 23.8 mm by misting). **Table 2** reports the mean air temperature measured close to the canopy during a representative heatwave week for each season. Data refers to the warmest period of the day, when the system was active, between 11:00 and 19:00 (from August 7th to 13th, 2024, and from August 8th to 14th, 2025; DOY 220-226). The misting system effectively reduced canopy air temperature and did so consistently across both seasons. On average, air temperature in the MI treatment was reduced by 1.3 °C in 2024, and by 5.9 °C in 2025. Despite a lower number of system activations in 2025 (approximately half of those recorded in 2024), the cooling effect was substantially greater (**Table 2**). To complete the microclimatic analysis of the cluster zone, the average vapor pressure deficit (VPD) was recorded at cluster level between 11:00 and 19:00 on the same day of the year. During the 2024 season, mean VPD values were 2.73 kPa in RC and 2.43 kPa in MI. In 2025, VPD values were higher in RC, reaching 3.44 kPa, compared to 1.68 kPa in MI (**Table 2**).

**Table 2:** Microclimatic conditions during DOY 220–225 in the 2024 and 2025 seasons (hr 11:00-19:00): mean air temperature within the RC canopy, mean air temperature within the MI canopy, difference between RC and MI mean air temperature, number of misting system activations and vapor pressure deficit (VPD).

	2024						2025					
DOY	RC (°C)	MI (°C)	Difference (°C)	Activations (N°)	VPD RC (kPa)	VPD MI (kPa)	RC (°C)	MI (°C)	Difference (°C)	Activations (N°)	VPD RC (kPa)	VPD MI (kPa)
220	34.40	33.46	-0.94	9	2.40	2.34	36.47	31.37	-5.01	4	3.17	2.04
221	32.45	31.74	-0.71	2	2.17	2.00	37.62	32.28	-5.34	5	3.41	2.05
222	34.31	32.98	-1.33	10	2.63	2.29	38.80	31.52	-7.28	8	4.17	1.69
223	34.75	33.91	-0.84	9	2.63	2.48	37.03	31.91	-5.12	5	3.22	1.65
224	35.71	34.05	-1.66	14	2.99	2.60	37.50	31.23	-6.27	5	3.40	1.48
225	36.69	34.74	-1.95	16	3.28	2.78	37.18	30.24	-6.94	5	3.64	1.37
226	36.08	34.61	-1.47	12	2.97	2.49	37.26	31.71	-5.55	4	3.41	1.48

**Figure 1:**
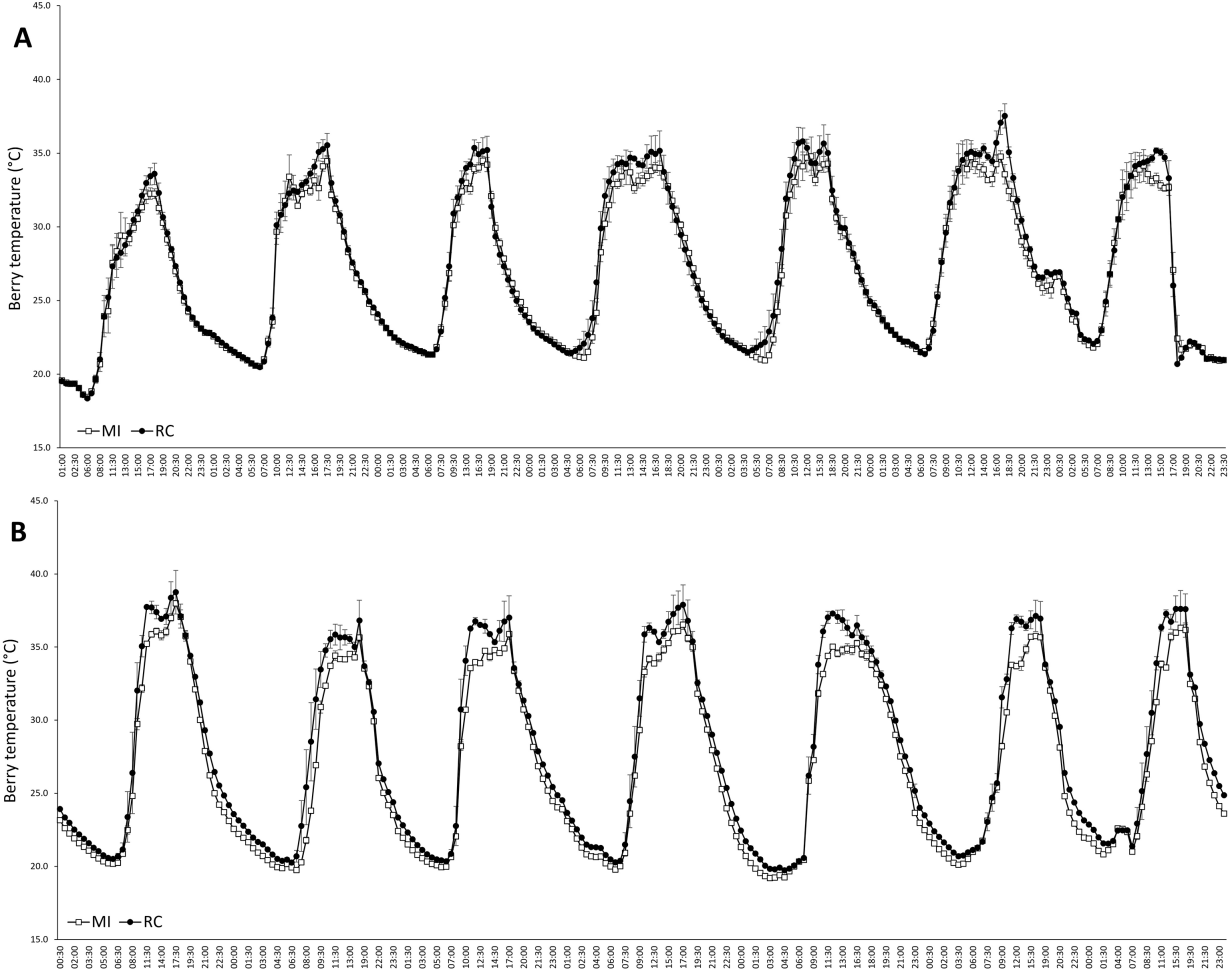
Berry temperature dynamics in MI and RC treatments during a representative heatwave week in August 2024 **(A)** and 2025 **(B)** (DOY 220–226). Each data point represents the mean of three thermocouples inserted into berries located on the east, west and central positions of the canopy. Error bars indicate standard error.

**Figure 1** shows the temporal patterns of berry temperature in MI and RC treatments during the representative heatwave week (DOY 220-226) in 2024 (A) and 2025 (B). In detail, berry temperatures were consistently lower in the MI treatment compared to RC following system activation, confirming the effectiveness of the misting system in reducing grape temperature throughout the day. The separation between MI and RC curves is evident in both seasons, with MI berries generally remaining below the threshold of 35 °C. A clear difference between years was observed, with 2025 (**Figure 1B**) showing markedly higher berry temperatures compared to 2024 (**Figure 1A**). In 2025, berry temperatures in the MI treatment remained consistently lower than those in RC, including during periods when the system was not active (e.g. nighttime).

### 3.3 Vine physiological responses

**Table 3** reports net photosynthesis (A), stomatal conductance (gs), and midday stem water potential (ψ) measured in MI and RC vines on three dates following system activation (DOY 206) during the 2024 season. Net photosynthesis increased in both treatments during August and declined after the hailstorm registered on August 27. Gas exchange parameters were generally higher in MI vines compared to RC, although significant differences were observed only on July 30 (DOY 212) when MI vines showed a +30% net photosynthesis compared to RC, while gs and ψ did not differ significantly. On DOY 220, stomatal conductance was significantly higher in MI vines, which also exhibited less negative stem water potential compared to RC (−5.65 vs −6.52 Bar), indicating a mild water deficit in the control treatment. On the same date, net photosynthesis also tended to be higher in MI vines, although the difference was only marginally significant (p = 0.068). Despite these differences, both treatments remained within mild to moderate stress conditions. By the final measurement DOY 248 (September 4), photosynthesis decreased in both treatments and no significant differences were observed for gs and ψ (**Table 3**).

**Table 3:** Assimilation rate (A), stomatal conductance (gs), and midday stem water potential (ψ) measured in MI and RC vines during the 2024 season.

	DOY 212			DOY 220			DOY 248		
Treatment	A (µmol CO2 m-² s-1)	gs (mol H2O m-² s-1)	ψ (Bar)	A (µmol CO2 m-² s-1)	gs (mol H2O m-² s-1)	ψ (Bar)	A (µmol CO2 m-² s-1)	gs (mol H2O m-² s-1)	ψ (Bar)
MI	16.64 a	0.322	-4.91	18.06	0.349 a	-5.65 a	14.83	0.300	-5.62
RC	12.76 b	0.304	-5.40	15.37	0.285 b	-6.52 b	14.80	0.367	-5.38
Significance	0.017	0.434	0.193	0.068	0.010	0.020	0.987	0.521	0.502

Measurements were taken on adult leaves of the main shoot. Different letters indicate significant differences between treatments within the same date (p ≤ 0.05).

In 2025, gas exchange was assessed on three occasions following the activation of the cooling system (**Table 4**). At the beginning of the season, gas exchange parameters were similar between treatments, indicating good leaf physiological performance. Differences between treatments became evident only at later stages. Stem water potential progressively decreased throughout the season, particularly in RC vines, reflecting the reduction of soil water availability. On DOY 216 (July 21), no significant differences were observed between treatments for net photosynthesis, stomatal conductance or stem water potential, with both treatments showing similar gas exchange rates and moderate water deficit conditions. At the second measurement (August 4), gas exchange parameters still did not differ significantly; however, stem water potential was significantly higher (less negative) in MI vines, likely reflecting the effect of post-veraison irrigation. Clear differences emerged on August 14 (DOY 226), following a period of six consecutive days with maximum temperatures exceeding 37 °C (**Table 2**). Under these conditions, MI vines exhibited significantly higher net photosynthesis and stomatal conductance, while RC vines showed markedly lower stem water potential (-10.01 Bar), indicative of severe water stress. In contrast, MI vines maintained higher water potential values consistent with moderate stress levels (**Table 4**).

**Table 4:** Assimilation rate (A), stomatal conductance (gs), and midday stem water potential (ψ) measured in MI and RC vines during the 2025 season. Measurements were taken on adult leaves of the main shoot.

	DOY 202			DOY 216			DOY 226		
Treatment	A (µmol CO2 m-² s-1)	gs (mol H2O m-² s-1)	ψ (Bar)	A (µmol CO2 m-² s-1)	gs (mol H2O m-² s-1)	ψ (Bar)	A (µmol CO2 m-² s-1)	gs (mol H2O m-² s-1)	ψ (Bar)
MI	11.19	0.244	-6.95	15.18	0.276	-5.09b	12.23a	0.244a	-7.00b
RC	11.13	0.226	-7.70	14.88	0.260	-7.47a	7.30b	0.099b	-10.01a
Significance	0.941	0.232	0.113	0.757	0.352	0.005	0.000	0.000	0.000

Different letters indicate significant differences between treatments within the same date (p ≤ 0.05).

### 3.4 Berry composition and berry weight dynamics during ripening

**Figure 2:**
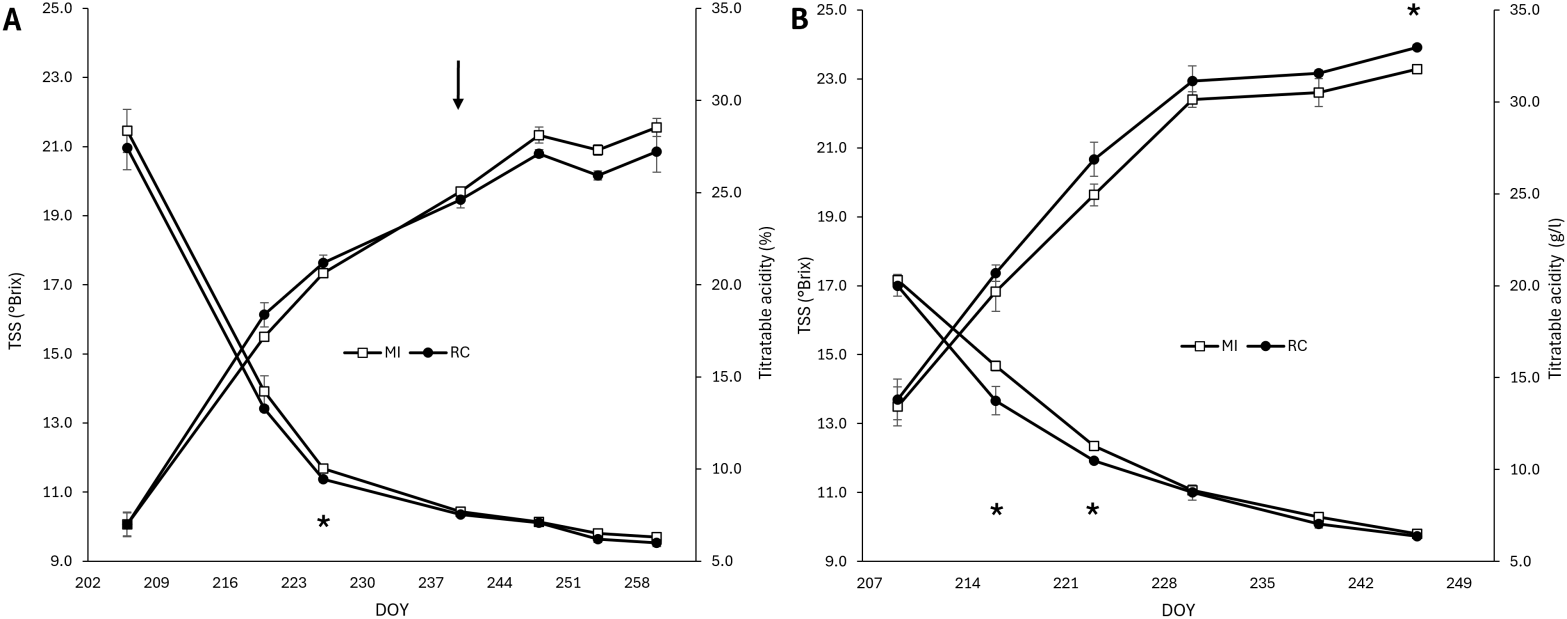
Evolution of Titratable acidity and Total soluble solids (TSS) in MI and RC treated vines from the 24th of July to the 16th of September 2024 [**(A)** DOY 206-260] and from July 28th to September 3rd 2025 [**(B)** DOY 209-246]. Error bars indicate the standard error. Asterisks (*) indicate significant differences between treatments according to ANOVA (p < 0.05). The arrow indicates the date of the hailstorm event (DOY 240).

**Figure 3:**
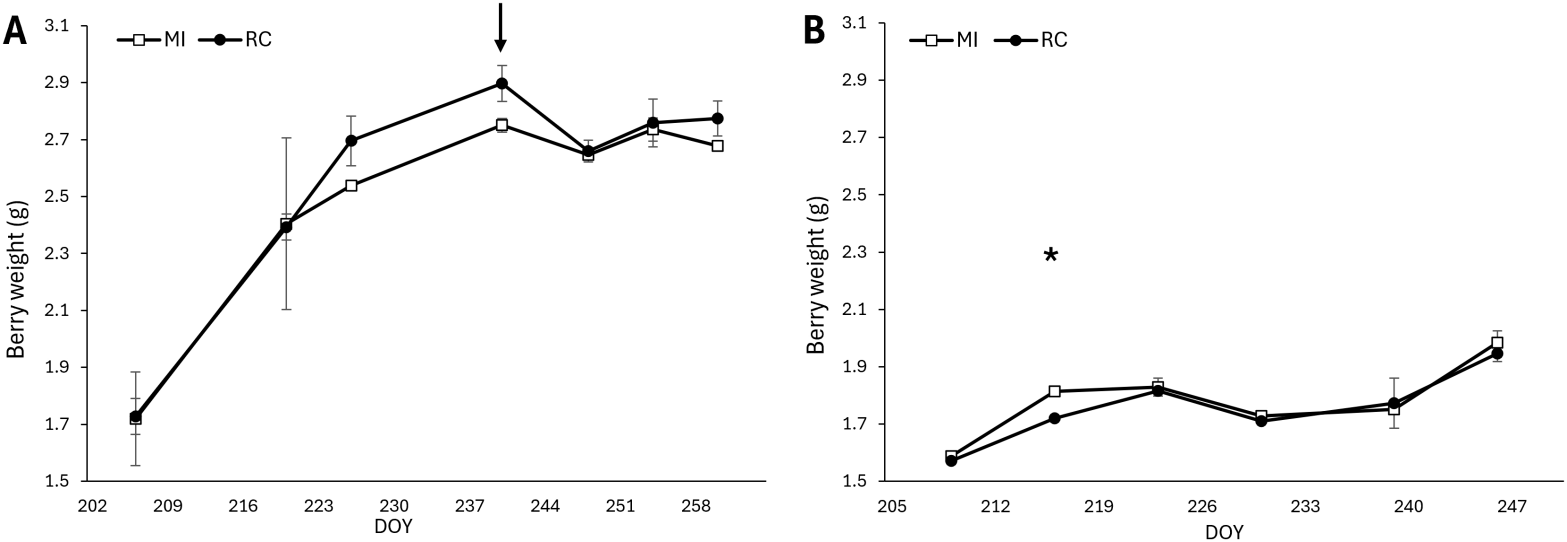
Evolution of berry weight in MI and RC treated vines from the 24th of July to the 16th of September 2024 [**(A)** DOY 206-260] and from July 28th to September 3rd 2025 [**(B)** DOY 209-246]. Error bars indicate the standard error. Asterisks (*) indicate significant differences between treatments according to ANOVA (p < 0.05). The arrow indicates the date of the hailstorm event (DOY 240).

Trends in total soluble solids (TSS) and titratable acidity (TA) during the 2024 and 2025 seasons are shown in **Figure 2**. In 2024, MI vines initially exhibited slightly higher titratable acidity and lower TSS compared to RC vines (**Figure 2A**). However, no significant differences were observed at harvest. This was likely due to the hailstorm event (DOY 240) and the exceptionally high rainfall recorded during summer (**Table 1**), with more than 60 mm accumulated between September 4th and 10th, effectively masking any treatment effects on technological maturity and berry mass (**Figure 3A**). In contrast, in 2025 MI vines consistently showed lower TSS compared to RC, with differences becoming significant at harvest (**Figure 2B**). Although the overall pattern of sugar accumulation was similar between treatments, MI vines exhibited a delayed accumulation of sugars. This delay was associated with a slower decline in titratable acidity, which does not appear to be related to dilution effects, as berry weight followed a similar trend in both treatments (**Figure 3B**).

**Figure 4:**
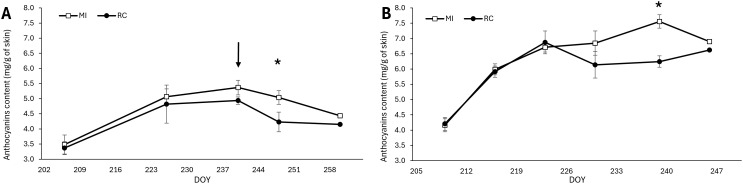
Trend of anthocyanins concentration (mg/g of skin) in MI and RC treated vines from the 24th of July to the 16th of September 2024 [**(A)** DOY 206-260] and from July 28th to September 3rd 2025 [**(B)** DOY 209-246]. Error bars indicate the standard error. Asterisks (*) indicate significant differences between treatments according to ANOVA (p < 0.05). The blue arrow indicates the date of the hailstorm event (DOY 240).

**Figure 4** shows the evolution of anthocyanin concentration in berry skins (mg/g) in MI and RC treatments during the 2024 and 2025 seasons. In 2024, MI vines consistently showed a tendency towards higher anthocyanin concentration compared to RC. Both treatments followed a similar seasonal pattern, with an initial increase followed by a decline after the hailstorm (**Figure 4A**; DOY 240). However, the decrease was more rapid in RC vines, whereas MI vines exhibited a more gradual decline, leading to a stabilization of pigment concentration towards the end of the season. In 2025, anthocyanin level was overall higher than in 2024 and showed a more dynamic pattern (**Figure 4B**). In MI vines, pigment accumulation increased progressively until the final measurement, followed by a slight decrease. In contrast, RC vines showed a less consistent trend, with an initial increase, a temporary decline, and a subsequent increase towards harvest. Notably, shortly before harvest, MI vines exhibited anthocyanin concentrations nearly 1 mg g^-^¹ higher than RC vines, and this difference was statistically significant, although both treatments converged to similar values at harvest. These differences were not associated with variations in berry weight, which did not differ significantly between treatments (**Figure 3B**).

### 3.5 Yield component and berry composition at harvest

**Figure 5:**
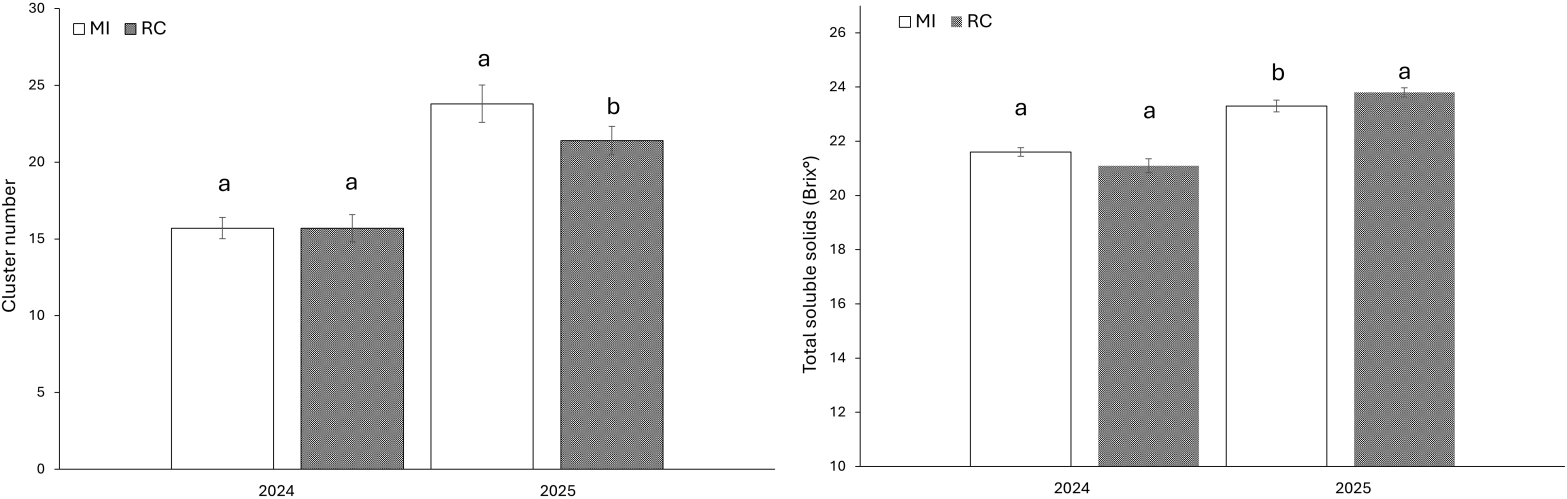
Number of clusters per vine and total soluble solids (TSS) in MI and RC treatments in 2024 and 2025. Bars represent mean values ± standard error. Different letters indicate significant differences between treatments within each year (p ≤ 0.05).

**Table 5** summarizes the effects of year (Y), irrigation (I), and their interaction (Y × I) on yield components and berry composition at harvest. Year significantly influenced most variables. In 2025, vines produced a greater number of clusters per vine, higher overall yield, and elevated total soluble solids (TSS) relative to 2024, while individual berry mass was significantly lower. Furthermore, grapes harvested in 2025 showed lower pH and higher titratable acidity, together with a marked increase in total anthocyanin concentration (**Table 5**). Irrigation, by contrast, had a limited effect on most parameters. No significant differences between irrigated (MI) and non-irrigated (RC) treatments were observed for yield, berry mass, TSS, or pH; titratable acidity, however, was significantly higher in MI vines. Sunburn incidence, evaluated only during the 2025 season, differed markedly between treatments. RC vines exhibited a sunburn incidence of 8.4%, whereas MI vines showed only 0.2%. Likewise, sunburn severity reached 45.2% of the exposed cluster area in RC vines but remained negligible (3%) in MI vines (**Supplementary Table 1**). A significant Year x Irrigation interaction (YxI) was detected for cluster number and TSS (**Figure 5**). As illustrated in **Figure 5**, no treatment differences were shown in 2024 season, whereas in 2025 MI vines presented a greater number of clusters and lower TSS compared to RC vines. These results indicate that multifunctional irrigation effects became detectable only under the climatic conditions of 2025. Pruning wood weight values are not reported in the table since no significant differences were observed between the two treatments. At the end of the growing cycle, MI showed a pruning wood weight of 0.9 kg compared to 0.8 kg in RC. Conversely, a significant difference was observed between vintages (p=0.000), with the wetter 2024 season showing higher values than the drier 2025 season, 1.02 kg and 0.7 kg respectively.

**Table 5:** Yield components and berry composition at harvest in the 2024 and 2025 growing seasons.

	Cluster (n)	Cluster weight (g)	Berry mass (g)	Yield (kg)	TSS (°Brix)	pH	Titratable acidity (g/L)	Total anthocyanins (mg/g of skin)
Year (Y)								
2024	16 ± 0.54	248 ± 8.1 9.2	2.72 ± 0.04	3.54 ± 0.18	21.4 ± 0.15	3.56 ± 0.01	6.16 ± 0.06	4.36 ± 0.21
2025	23 ± 0.88	219 ± 9.2	2.00 ± 0.04	5.14 ± 0.29	23.6 ± 0.15	3.46 ± 0.01	6.44 ± 0.09	6.63 ± 0.14
p-value	0.000	0.583	0.000	0.021	0.000	0.000	0.012	0.000
Irrigation (I)								
MI	20 ± 1.35	236 ± 9.6	2.34 ± 0.07	4.44 ± 0.28	22.4 ± 0.20	3.50 ± 0.01	6.41 ± 0.08	5.57 ± 0.25
RC	18 ± 0.90	231 ± 7.8	2.37 ± 0.09	4.23 ± 0.27	22.5 ± 0.20	3.51 ± 0.01	6.19 ± 0.08	5.42 ± 0.30
p-value	0.120	0.996	0.595	0.540	0.775	0.778	0.043	0.189
Y x I	0.047	0.356	0.162	0.997	0.021	0.355	0.426	0.132

Values are presented as means ± standard error (SE). The effects of year (Y), irrigation (I), and their interaction (YxI) are reported. Statistical significance was assessed by ANOVA and p-values are presented for main factors and their interaction.

## 4 Discussion

This study aimed to evaluate, over two growing seasons (2024-2025), the effects of a combined post-veraison irrigation and fruit-zone cooling strategy on vine water status, berry ripening dynamics and phenolic composition in the red cultivar Sangiovese (*Vitis vinifera* L). Two treatments were compared: a multifunctional irrigation system (MI), combining drip irrigation and evaporative cooling, and a non-irrigated control (RC). It should be emphasized, however, that the irrigation strategy adopted in the present study should not be considered equivalent to conventional vineyard drip irrigation, since water was supplied only from veraison onward to compensate vine transpiration demand, whereas evaporative cooling was activated only when critical temperature thresholds were exceeded. The two seasons were characterized by markedly different climatic conditions. The 2024 season was unusually wet and affected by a severe hailstorm, whereas 2025 was hotter and drier during the ripening period. Due to the substantial damage caused by hail in 2024, which affected both canopy and fruit development, the interpretation of treatment effects in that season is limited. Consequently, greater emphasis is placed on 2025, where climatic conditions allowed a clearer assessment of treatment responses. Under these contrasting conditions, the multifunctional cooling-irrigation system showed a markedly different efficiency between seasons. Although these findings provide valuable insight into the response of Sangiovese under the environmental conditions of a flatland vineyard, caution is required when extrapolating these results to other cultivars and viticultural environments, as the magnitude of the response is likely to depend on vineyard architecture and local environmental conditions.

The contrasting climatic conditions between the two seasons strongly influenced vine water relations and the performance of the irrigation-cooling system. In 2024, the high rainfall recorded during the ripening period, particularly close to harvest, resulted in limited water input from the drip irrigation system. Under these conditions, soil water availability was not limiting, and evapotranspiration was not constrained, as reflected by the similar evaporative demand (VPD) observed between treatments. In contrast, the drier and warmer conditions of 2025 led to a more pronounced soil water deficit, particularly in RC vines, while increasing atmospheric evaporative demand. As a result, irrigation played a key role in sustaining vine water status, although overall transpiration remained constrained by soil water availability, as indicated by the lower seasonal evapotranspiration compared to 2024. These conditions also affected the efficiency of the cooling system. The multifunctional irrigation consistently reduced air and berry temperature in the cluster zone under both seasonal conditions, confirming its effectiveness as a cooling strategy. Berry temperatures in MI vines remained significantly lower than in the control during the warmest periods of the day, in agreement with previous studies on evaporative cooling systems in viticulture (Greer and Weedon, 2016; Caravia et al., 2017; Bianchi et al., 2023; Valentini et al., 2024; Bonini et al., 2025a).

The greater cooling effect observed in 2025, despite a lower number of system activations, was likely driven by higher evaporative demand. In particular, the increased VPD in the cluster zone enhanced water evaporation and latent heat dissipation, resulting in a more pronounced cooling effect (Jones, 2014). Conversely, the more humid conditions in 2024 likely limited evaporation, reducing misting efficiency despite more frequent activations (Allen et al., 1998). Interestingly, in 2025 the MI treatment exhibited an earlier decline in berry temperature during the evening, even after the cooling system was no longer active (**Figure 1B**). This suggests a carry-over effect of daytime microclimatic conditions, whereby lower temperature and higher humidity in the bunch zone reduced heat storage and promoted faster thermal dissipation. In addition, the improved plant water status under MI may have enhanced the thermal buffering capacity of berry tissues, further contributing to faster cooling dynamics (Jones, 2014; Gambetta et al., 2020).

The physiological data collected during both seasons confirm that the combined irrigation-cooling system improved vine water status and maintained leaf gas exchange activity during peak stress periods. In 2024, differences between treatments were moderate, consistent with the abundant rainfall that reduced the effective water deficit in RC vines. Nevertheless, MI vines showed significantly higher net photosynthesis early in the season in DOY 212 and in DOY 220 (p=0.068), as well an increase in stomatal conductance during the heatwave period (DOY 220), together with less negative stem water potential (**Table 3**). These findings are consistent with the general understanding that even mild improvements in vine water status can sustain stomatal aperture and carbon assimilation under moderate stress (Flexas et al., 2014). Similar results were detected by Bonini et al. (2025b), who reported an improvement in canopy efficiency even when the system directly acts on the cluster zone. In 2025, the physiological advantage of the MI treatment became far more evident. Following a prolonged heatwave with six consecutive days exceeding 37 °C, RC vines reached a stem water potential of -10.01 Bar, indicative of severe water stress (**Table 4**). Under these conditions, stomatal conductance and photosynthesis in RC vines declined sharply, while MI vines maintained significantly higher values for all three parameters. These results align with previous findings on post-veraison irrigation in grapevine, where supplemental water supply during ripening prevented the decline in leaf physiological performance typically associated with late-season drought (Dinis et al., 2018; Allegro et al., 2023; Valentini et al., 2024). Importantly, the cooling component of the system likely contributed to sustaining stomatal conductance by reducing canopy temperature and, consequently, leaf-to-air vapor pressure deficit, thereby lowering evaporative demand at the leaf level (Jones, 2014).

These physiological responses at the leaf level likely had downstream effects on berry ripening dynamics. In particular, the combined reduction in canopy temperature and evaporative demand may have altered the microclimatic and hydraulic conditions surrounding the clusters, thereby influencing sugar accumulation patterns. One of the most relevant findings of this study is the significant delay in total soluble solids (TSS) accumulation observed in MI vines during the 2025 season. The YxI interaction detected for TSS (p = 0.021; **Table 5**) indicates that the irrigation-cooling treatment effectively slowed sugar accumulation, but only under the hotter and drier conditions of 2025. In 2024, the abundant summer rainfall masked treatment effects on berry composition, as both MI and RC vines reached comparable TSS levels at harvest. In contrast, in 2025 MI vines consistently displayed lower TSS throughout the ripening period, with significant differences at harvest (**Figure 2B**). This delay in sugar accumulation was not attributable to a dilution effect, since berry weight did not differ significantly between treatments (**Figure 3B**). Rather, the slowdown in TSS is more likely linked to the reduced canopy and berry temperatures achieved through misting, which may have directly lowered the rate of metabolic processes driving sugar accumulation. Temperature is a key driver of sugar accumulation during grape ripening and contributes to the decoupling between technological and phenolic maturity frequently observed under warm climatic conditions (Palliotti et al., 2014; Valentini et al., 2019). Moderate warming generally accelerates sugar accumulation, leading to wines with higher alcohol content and altered sensory balance (Mira de Orduna, 2010; Keller, 2010), whereas excessive heat may impair ripening processes (Greer and Weston, 2010; Greer and Weedon, 2013; Palliotti et al., 2014). Consequently, the ability of the multifunctional system to delay sugar accumulation is of considerable practical relevance, as it may allow harvest to be postponed while limiting excessive sugar levels and preserving phenolic development.

Our findings are consistent with those of Bonini et al. (2025b), who reported that canopy cooling not only reduced cluster temperature but also preserved the functionality of upper canopy leaves, mitigating atmospheric water stress and maintaining leaf physiological performance. Although the MI system used in the present study differed substantially from that described by Bonini et al. (2025b), mainly because of the considerably lower amount of water applied through misting, both studies showed improved leaf physiological performance associated with delayed technological ripening. In this regard, the effect observed in the present study is consistent with the finding of Filippetti et al. (2011), who reported that post-veraison trimming could slow sugar accumulation in Sangiovese without modifying phenolic ripening, and extends this concept by showing that a similar outcome can be achieved through environmental manipulation of the cluster microclimate.

The observed reduction in bunch-zone VPD and air temperature under the misting treatment, indicates that the response was primarily driven by modifications of the cluster microclimate rather than vine water status per se. In line with Zhang and Keller (2015), berry transpiration is largely cuticular and passively driven by VPD, with limited capacity for active regulation. Therefore, the lower evaporative demand under MI conditions likely altered berry heat balance and slowed sugar accumulation. Consistently, berry transpiration has been shown to be strongly controlled by atmospheric conditions and to play a key role in ripening dynamics (Gambetta et al., 2020). From a mechanistic perspective, reduced transpiration leads to a more positive berry water balance and lower solute concentration, ultimately limiting sugar accumulation (Zhu et al., 2019). This supports the hypothesis that the lower TSS observed under misting conditions was primarily driven by reduced transpiration associated with lower VPD and temperature.

However, cultivar-specific responses must also be considered. In contrast to the findings of Cabodevilla et al. (2024) in Tempranilllo and Martinez-Lüscher et al. (2025) in Malbec, where reduced berry transpiration through the use of anti-transpirants and shading nets led to decreased flavonoid accumulation, Filippetti et al. (2011) reported a different response in Sangiovese. In their study, the application of a pinolene-based antitranspirant after veraison reduced sugar accumulation without significantly affecting anthocyanin concentration. In line with these observations, the present results indicate that, in Sangiovese, technological maturity may be more sensitive than phenolic development to changes in berry transpiration and microclimate. In detail, the observed differences in anthocyanin dynamics between MI and RC treatments suggest distinct patterns of biosynthesis and degradation during ripening. In 2024, both treatments followed a similar temporal evolution, although MI vines maintained slightly higher anthocyanin levels and exhibited a slower decline following the hailstorm event. In 2025, the divergence between treatments was more pronounced, with MI vines reaching sensibly higher anthocyanin concentrations prior to harvest, whereas RC vines showed a more variable pattern, including a temporary decline followed by a late-season increase (**Figure 4B**). These differences may be related to the contrasting vine water status observed between treatments. RC vines experienced a more pronounced late-season water deficit, which is known to stimulate anthocyanin biosynthesis in red grape cultivars (Castellarin et al., 2007), potentially explaining the increase in pigment concentration close to harvest. Conversely, MI vines maintained a more favorable water status during ripening, resulting in more stable anthocyanin accumulation and a slower decline towards the end of the season. This behavior suggests that, under reduced water and thermal stress conditions, anthocyanin concentration may be preserved not only through sustained biosynthesis but also through limited degradation induced by higher temperature (Movahed et al., 2016). The lower berry temperatures observed in MI vines during the warmest period likely contributed to this effect, as high temperatures are known to enhance anthocyanin degradation in addition to limiting their biosynthesis (Mori et al., 2007; Movahed et al., 2016). These findings are consistent with previous studies on intra-canopy cooling systems, where limited differences in anthocyanin concentration were observed between treated and control vines (Caravia et al., 2017; Kliewer and Schultz, 1973), supporting the hypothesis that manipulating bunch-zone microclimate can partially re-couple sugar accumulation and anthocyanin development by slowing technological ripening without compromising phenolic maturity.

The marked reduction of sunburn damage in MI vines represents another key outcome of the multifunctional irrigation system. In 2025, RC vines exhibited a sunburn incidence of 8.5% on harvested clusters, whereas no damage was recorded in the irrigated-cooled treatment (**Table 5**). The 2025 results clearly demonstrate the protective capacity of the system under field conditions. Sunburn in grapevine results from the combined effects of high solar radiation and elevated berry surface temperature, which can exceed critical thresholds causing tissue necrosis and oxidative damage to phenolic compounds (Allegro et al., 2025). By maintaining berry temperatures consistently below 35 °C during peak radiation hours (**Figure 1B**), the misting system effectively prevented the occurrence of sunburn symptoms. This result is consistent with findings from previous studies on the same cooling system applied to potted Sangiovese vines (Valentini et al., 2024), and to the red and white cultivar Barbera, Sauvignon and Pignoletto under field conditions (Bonini et al., 2025a; Valentini et al., 2025), where evaporative cooling significantly reduced sunburn incidence and preserved yield integrity. Unlike shading nets or foliar particle films, which reduce radiation at the expense of light interception and may affect flavonoid biosynthesis (Oliveira et al., 2014; Teker, 2023; Allegro et al., 2024), evaporative cooling acts selectively on temperature without altering the radiation regime. This specificity makes it particularly suitable for red cultivars such as Sangiovese, where light exposure is essential for adequate anthocyanin and flavonol accumulation (Pastore et al., 2017; Romboli et al., 2017).

A further highly positive effect of the temperature reduction in MI is reflected in the acid balance of the berries at harvest. The higher titratable acidity recorded in MI vines across both seasons (**Table 5**) further supports the interpretation that the combined irrigation–cooling system attenuated ripening dynamics. Organic acid degradation during berry ripening is temperature-dependent, with malic acid in particular being rapidly metabolized under warm conditions (Rienth et al., 2016). The lower cluster temperatures recorded in MI vines likely slowed malic acid respiration, thereby maintaining higher acidity levels at harvest. This response is particularly relevant under warm ripening conditions, where accelerated malic acid degradation frequently leads to musts with excessively low acidity, often requiring corrective oenological interventions (Van Leeuwen et al., 2022). Notably, the maintenance of higher titratable acidity in MI vines occurred alongside a delay in TSS without a significant reduction in anthocyanin concentration at harvest. This pattern contrasts with previous studies reporting a simultaneous delay in TSS and anthocyanin accumulation under post-veraison irrigation (Theocharis et al., 2021). In the present study, the anthocyanin concentration at harvest did not differ significantly between treatments (**Table 5**), suggesting that the cooling component of the system may have partially offset the dilution or inhibitory effects typically associated with post-veraison water supply (Ojeda et al., 2002; Castellarin et al., 2007). In this perspective, the combined strategy appears to slow down technological maturity while preserving phenolic maturity, a result that has been identified as one of the main challenges in warm-climate viticulture (Palliotti et al., 2014; Valentini et al., 2019).

In conclusion, a significant Yx I interaction was also observed for cluster number per vine, with MI vines producing more clusters than RC only in 2025. Although cluster number is typically determined before veraison, this difference may reflect the higher incidence and severity of sunburn observed in RC vines. In 2025, RC vines exhibited an 8.5% sunburn incidence, with damage affecting up to approximately 50% of the exposed cluster area. Because completely desiccated clusters were excluded from yield determination, severe sunburn may have contributed to the lower number of harvested clusters recorded in the RC treatment.

## 5 Conclusions

The results of this two-year field study suggest that a multifunctional irrigation system combining post-veraison drip irrigation with fruit-zone evaporative cooling may represent a promising climate change adaptation strategy for warm-climate viticulture. Under the hot and dry conditions of 2025, the system significantly reduced air and berry temperature, resulting in measurable physiological and agronomic benefits. In particular, MI vines maintained higher stem water potential, stomatal conductance, and photosynthetic activity during peak stress periods, indicating that the combined strategy effectively preserved leaf physiological functionality under severe thermal conditions.

One of the most relevant outcomes of the study was the significant delay in total soluble solids (TSS) accumulation without a concurrent increase in berry size, indicating that the slowdown was driven by attenuated metabolic rates under cooler berry temperatures rather than by a simple dilution effect. At the same time, the moderate divergence in TSS between treatments suggests that the delay in ripening was partially compensated by the higher photosynthetic efficiency maintained in MI vines. This outcome is of direct practical relevance because it contributes to mitigating the premature attainment of technological maturity before adequate phenolic development, one of the major challenges of warm-climate viticulture.

From the perspective of berry composition, the combined strategy proved capable of preserving anthocyanin concentration at harvest despite the provision of supplemental water during ripening, a condition that, when applied as irrigation alone, has been repeatedly associated with pigment dilution or biosynthetic inhibition. The lower berry temperatures achieved through misting appear to have limited anthocyanin oxidative degradation during the warmest phase of the season, as reflected in the higher pigment concentration observed before harvest. At the same time, titratable acidity was significantly higher in treated vines, consistent with reduced malic acid catabolism under cooler fruit-zone conditions. Taken together, these responses indicate that the system can slow technological maturity while maintaining anthocyanin integrity and acidity, effectively mitigating the decoupling that characterizes warm-climate ripening.

These results suggest that, under the environmental conditions of the present study, grapes could potentially be harvested at lower sugar concentrations while maintaining anthocyanin concentration, although confirmation through dedicated vinification trials is still required.

The present findings also suggest that similar physiological and ripening responses to those previously reported with nebulization systems in red cultivars can be achieved while functionally separating irrigation and cooling management.

The significant YxI interactions detected for both TSS and cluster number underscore, however, that the benefits of the system are contingent upon seasonal climatic severity: in the wet 2024 season, treatment effects were largely masked by abundant rainfall and reduced evaporative demand. This seasonal dependency should not be regarded as a limitation but rather as evidence that the system functions as an adaptive, demand-driven tool whose contribution scales with the intensity of environmental stress.

The agronomic relevance of the proposed strategy was most clearly demonstrated under the hot and dry conditions of the 2025 season, whereas the unusually wet 2024 season largely masked treatment effects. Furthermore, the present outcomes were obtained in a single flatland Sangiovese vineyard under relatively moderate drought conditions. Therefore, additional multi-season studies are needed to evaluate the performance of the system across different cultivars, vineyard architectures and topographical conditions, particularly in hillside vineyards and under more severe drought and heat stress, before broad recommendations can be made.

## Data Availability

The raw data supporting the conclusions of this article will be made available by the authors, without undue reservation.
