# Supplementary material for: Multifunctional irrigation mitigates heat stress, delays sugar accumulation and preserves berry composition in cv. Sangiovese (Vitis vinifera L.)
Source: Front Plant Sci. 2026 Aug 5;17:1911234. doi: 10.3389/fpls.2026.1911234 (PMC13487748; doi:10.3389/fpls.2026.1911234)
Supplement: Supplementary file 1 [file Table1.docx]

**13. Supplementary materials**

**Table S1.** Sunburn incidence and severity assessed at harvest during the 2025 growing season. Values represent treatment means. Sunburn incidence was analyzed using a generalized linear mixed model (GLMM), whereas sunburn severity was analyzed only on vines exhibiting sunburn symptoms after Bliss angular transformation using one-way ANOVA.

|  | Sunburn Incidence (%) | Sunburn Severity (%) |
| --- | --- | --- |
| MI | 0.2 | 3 |
| RC | 8.4 | 45.2 |
| *P-value* | ***0.0076*** | ***0.000*** |
